# Impact of yogurt consumption on bone health markers in adults with or without osteoporosis: a systematic review and meta-analysis

**DOI:** 10.3389/fnut.2025.1660505

**Published:** 2025-09-30

**Authors:** Baltasar Mayo, Jeadran Malagón-Rojas, Fojan Agahi, Juana Frias, İbrahim Ender Künili, Mary-Liis Kütt, Julie Mardon, Daniela Nikolovska Nedelkoska, Mayra Alejandra Parada, Aleksandra Torbica, Tuğçe Bulmuş-Tüccar, Birsen Yilmaz, Cornelia Bär, Hayriye Sebnem Harsa, Christophe Chassard, Smilja Praćer, Guy Vergères, Jyoti Prakash Tamang

**Affiliations:** ^1^Instituto de Productos Lácteos de Asturias (IPLA-CSIC), Oviedo, Spain; ^2^Investigación Sanitaria del Principado de Asturias (ISPA), Oviedo, Spain; ^3^Observatorio Nacional de Salud, Instituto Nacional de Salud, Bogotá, Colombia; ^4^Instituto de Ciencia y Tecnología de Alimentos y Nutrición (ICTAN-CSIC), Madrid, Spain; ^5^Department of Fishing and Fish Processing Technology, The Faculty of Marine Sciences and Technology, Çanakkale Onsekiz Mart University, Çanakkale, Türkiye; ^6^Äio tech OÜ, Tallinn, Estonia; ^7^Université Clermont Auvergne, INRAE, VetAgro Sup, Lempdes, France; ^8^Faculty of Technology and Technical Sciences, University “St. Kliment Ohridski”-Bitola, Veles, Republic of North Macedonia; ^9^Grupo de Salud Ambiental y Laboral, Instituto Nacional de Salud, Bogotá, Colombia; ^10^Institute of Food Technology, University of Novi Sad, Novi Sad, Serbia; ^11^Department of Nutrition and Dietetics, Yüksek Ihtisas University, Ankara, Türkiye; ^12^Department of Biological Sciences, Tata Institute of Fundamental Research, Hyderabad, India; ^13^Agroscope, Bern, Switzerland; ^14^Department of Food Engineering, İzmir Institute of Technology, İzmir, Türkiye; ^15^UCA, INRAE, VetAgro Sup, Aurillac, France; ^16^Institute for Biological Research Siniša Stanković, National Institute of the Republic of Serbia, University of Belgrade, Belgrade, Serbia; ^17^Department of Microbiology, Sikkim University, Gangtok, India

**Keywords:** yogurt, fermented food, bone health, osteoporosis, osteopenia, systematic review, meta-analysis

## Abstract

Yogurt is a fermented dairy product widely recognized for its nutritional value and potential health benefits, particularly due to its high content of calcium, phosphorus, vitamins, and other bioactive compounds. Its purported role in promoting bone health has attracted increasing attention, especially among adults at risk of osteoporosis. However, the specific impact of yogurt consumption on bone health remains controversial, as current evidence is limited and often inconclusive. This systematic review and meta-analysis sought to evaluate the exclusive effects of yogurt consumption on bone health in adults with and without osteoporosis. Employing rigorous inclusion and exclusion criteria and standardized methodologies, the study reviewed data from both observational and clinical studies to assess the effect of yogurt consumption on bone mineral density (BMD), fracture risk, and other bone health markers. In addition, yogurt characteristics were documented in accordance with EFSA guidelines, the possible bioactive components listed, and their potential mechanism of action in relation to bone health discussed. Studies fitting the inclusion and exclusion criteria (*n* = 12) were all observational, limiting the ability to infer causality. The meta-analysis of cohort studies (*n* = 6) found no significant association between yogurt consumption and hip fracture risk (HR = 1.01, 95% CI: 0.96–1.07), while the observed positive effect on BMD was minimal and clinically irrelevant (SMD = 0.009). Although some studies reported modest improvements in BMD and bone biomarkers, the overall evidence remains inconclusive, hindered by the heterogeneity in study designs and inconsistent yogurt intake. In conclusion, current evidence does not support a significant role of yogurt consumption in preventing fractures or improving BMD in adults. Well-designed randomized controlled studies are needed to clarify its effects, particularly in adults at risk of or with osteoporosis or osteopenia. In conclusion, the effectiveness of yogurt as a primary strategy for enhancing bone health is not fully substantiated by current data, though its inclusion within a balanced diet may still offer benefits for bone health.

**Systematic review registration:**

https://osf.io/, 10.17605/OSF.IO/ES2PM

## 1 Introduction

Yogurt is defined by the Codex Alimentarius as “a coagulated dairy product obtained through the fermentation of milk by the symbiotic action of *Streptococcus thermophilus* (*S. thermophilus*) and *Lactobacillus delbrueckii* subsp. *bulgaricus* (*L. bulgaricus*)” (1). Although the biochemical composition of milk and yogurt is highly similar, yogurt offers unique nutritional advantages due to the metabolic activities of *S. thermophilus* and *L. bulgaricus*. These bacteria can synthesize vitamins –particularly those from groups B and K– and generate compounds with potential biological activity during fermentation (e.g., lactic acid, γ-aminobutyric acid, and bioactive peptides) (2–4). The bacteria in yogurt must be alive, which is essential to the product’s identity and potential health benefits. Despite its standard definition, yogurt presents considerable diversity in terms of composition and processing. It can be prepared from milk of various species, and is available in multiple forms –such as plain, semi-skimmed or skimmed milk–, depending on regional traditions and production practices. Beyond the high nutritional value of milk, yogurt is endowed with additional beneficial effects, such as enhancing lactose tolerance, reducing the risk of diabetes type 2, improving digestive health, immune function, metabolic health, mental well-being, and bone health (4–9). In agreement with these benefits to human health, yogurt has been endowed with a health claim issued by the European Food Safety Agency (EFSA) in 2010 which states that “live yogurt cultures can improve lactose digestion in individuals with lactose maldigestion” (10) and, more recently, with a Federal Drug Agency health claim that states that “eating yogurt regularly may reduce the risk of type 2 diabetes” (11). Beneficial assumptions and health claims have supported the inclusion of yogurt in many dietary recommendations and guidelines.

Bone health refers to the overall condition of bone formation, maintenance and repair during the whole human lifespan (12). Bone formation is a crucial process that occurs mainly during childhood and adolescence, which results in an increase in bone mass and bone strength. Nonetheless, bone is a living tissue that is constantly undergoing formation, remodeling and repair. In the adulthood (from 30 years onward) bone formation slows down and bone resorption increases. This may lead to a gradual loss of bone mass (12), which can end up in the adulthood with fragility fractures (e.g., hip, forearm, and vertebrae), a sign of underlying bone conditions such as osteopenia or osteoporosis. Calcium, phosphorous, and vitamin D are considered essential compounds in bone physiology and health (13). Absorption of minerals in the intestine is stimulated by vitamin D. This vitamin is responsible for the abortion of more than 90% of the calcium (14). Association studies have further identified vitamins of the groups B and K as pivotal dietary factors in bone health (15, 16). To meet the specific requirements of human life’s stages and personal conditions, all these nutrients have to be ingested in enough quantity and in a bioavailable form (12).

Yogurt is a good source of high-quality protein, calcium, potassium, magnesium, phosphorus, zinc, selenium, vitamin A, and vitamins of the group B and K (4), of which many are known to enhance bone growth and health (17). The enhanced ionization of minerals and the increased amounts of vitamins B and K in yogurt as compared to milk has hypothesized that yogurt consumption provides a means to maintain and enhance bone health. However, the current scientific evidence on the effect of yogurt consumption on bone health in adults remains controversial, as both positive (18–21) and neutral (22–27) associations have been reported in different studies. Despite the disagreement or variability in findings across individual studies, it remains crucial to pool the data together to derive a comprehensive and aggregated outcome. Systematic reviews and meta-analysis have the potential to quantitatively synthesize the evidence when disagreement between studies in a given topic persists (28). However, scarce studies using a systematic review approach exist on yogurt consumption and bone health (9). Instead, the role of yogurt consumption on bones has usually been investigated grouped under the umbrella of milk, dairy or fermented dairy products (29–33). This highlights the importance of conducting a systematic review to address the existing gaps and provide a consolidated understanding of yogurt consumption’s role in bone health.

In this context, and within the framework of the PIMENTO Working Group 3 (WG3) initiative (34), the present study aimed to conduct a comprehensive systematic review to evaluate the effect of yogurt consumption on bone health in adults with or without osteoporosis. A distinctive feature of this review is its integrative approach, which not only synthesizes human clinical evidence but, in accordance with EFSA’s guidance, also includes dedicated sections on Yogurt Characterization, Bioavailability of Bioactive Compounds, and Mechanism of Action. This structure allows for a more in-depth analysis of exposure, biological plausibility, and potential variability across studies, setting it apart from conventional systematic reviews.

## 2 Methods

This review was carried out by subgroup E6 of PIMENTO Working Group 3 (WG3). A study protocol was written and approved by members of WG3 of the PIMENTO COST Action. The protocol was registered in the Open Science Framework (OSF)^1^ under the identifier doi: 10.17605/OSF.IO/ES2PM.

### 2.1 Systematic review of human studies

This systematic review was conducted following the methodological standards of the Cochrane Handbook Systematic Reviews of Interventions (35) and adhered to the PRISMA 2020 statement (36) to ensure transparent and comprehensive reporting. The planning, coordination, iterative updates, and evidence synthesis followed the structured approach proposed by Muka et al. (37) and EFSA guidance (38, 39), with adaptations based on the PIMENTO Study Protocol. The EFSA guidance was followed specially to incorporate food characterization, mechanism of action, safety and mechanistic substantiation and evidence grading, which was included in our review as a part of the non-systematic analysis described in Section “2.2 Non-systematic components of the review.”

#### 2.1.1 Literature search

A comprehensive systematic literature search was conducted in three electronic databases: PubMed, Scopus, and the Cochrane Library, covering publications from January 1, 1970 to October 31, 2023. A search update from November 1, 2023 to December 31, 2024 was done in PubMed. Only articles published in English were considered. The search strategy employed the generic PIMENTO search strings covering fermented foods, dietary intake, and human clinical outcomes, complemented with review-specific keywords including “osteoporosis,” “bone health,” “bone mineral density,” “osteopenia,” and “fractures.” No modifications were made to the generic PIMENTO search string structure beyond the inclusion of these bone health-related terms. The complete search strings are presented as Supplementary material in Supplementary Table 1; they were published in our project on open science framework repository.^2^ The study selection process was documented using a PRISMA flow diagram.

#### 2.1.2 Eligibility criteria

Inclusion and exclusion criteria (Table 1) were established according to the PICO framework (40):

**TABLE 1:** PICOS criteria for inclusion and exclusion of studies.

Element	Inclusion and exclusion criteria for studies
Population	Adults aged 18 years and older, with or without a clinical diagnosis of osteoporosis (confirmed by bone density scan). Studies with pregnant or lactating women were excluded.
Intervention/exposure	Ingestion of yogurt as part of dietary consumption or as a nutritional intervention. Eligible yogurt products included those naturally fermented with the traditional starter cultures, strains of *S. thermophilus* and *L. delbrueckii* subsp. *bulgaricus*. The occasional presence of additional probiotics cannot be discarded. Interventions containing confounders such as prebiotic fibers or added bioactive compounds beyond those naturally present in yogurt were excluded.
Comparator	No yogurt consumption, placebo (e.g., chemically acidified milk), non-fermented dairy counterparts, or standard osteoporosis treatments (e.g., calcium plus vitamin D supplementation, bisphosphonates). Studies comparing only different fermented products without an appropriate control group were excluded. To assess potential dose-response relationships, comparisons were made in observational studies between different yogurt intake levels (e.g., daily vs. weekly consumption, tertiles, quartiles, etc.).
Outcomes	Primary outcomes included changes in bone mineral density, transition in bone status (e.g., from osteoporosis to osteopenia), incidence of pathological fractures (hip, spine, wrist), serum calcium and alkaline phosphatase levels, and bone turnover markers. Quality of life measurements, body mass index, and reported adverse effects.
Study design	Randomized controlled trials, non-randomized intervention studies, and observational studies (cohort, case-control, cross-sectional) were included. Available systematic reviews were screened for potentially missing primary studies. *In vitro* studies and animal trials were excluded from the systematic review process.

#### 2.1.3 Study selection

Study screening and data extraction followed the methodology outlined by Muka et al. (37) in steps 4 through 18. Titles and abstracts were independently screened by two reviewers using CADIMA software (41). Duplicate records were removed during the initial screening phase. All the researchers were trained in identifying eligibility criteria during the screening phases (titles, abstracts, and full texts). Agreement was measured using Kappa index in CADIMA. The screening process began only after the reviewers achieved a high level of agreement (Kappa = 0.8) in both the title/abstract and full text screening phases, ensuring consistency and reliability in study selection. Full-text articles that passed the preliminary screening were assessed against the complete eligibility criteria. Disagreements between reviewers were solved through consensus discussion or, when necessary, judged by a third reviewer.

#### 2.1.4 Data extraction

A standardized data extraction form was developed based on guidance from the Cochrane Handbook for Systematic Reviews of Interventions (35), EFSA’s Appendix B for human efficacy studies (39), and the STROBE checklist for observational studies (42). Sheets in Google were used to extract data. Each full text article was reviewed at least by two reviewers. Extracted data were compared and discussed. Data extracted included: study design characteristics, participant demographics, yogurt composition and dosage, intervention duration and exposure for observational studies, comparator details, outcome measurement methodologies, and numerical results with measures of variability.

#### 2.1.5 Risk of bias assessment

The Newcastle-Ottawa Scale (43) was applied, evaluating selection, comparability, and outcome assessment. Two reviewers independently evaluated each study, with discrepancies resolved by consensus or third-party adjudication.

The certainty of evidence across studies was evaluated using the Grading of Recommendations Assessment, Development and Evaluation (GRADE quality evaluation) approach (44), rating evidence as high, moderate, low, or very low based on risk of bias, inconsistency, indirectness, imprecision, and publication bias.

#### 2.1.6 Data synthesis and analysis

Data synthesis followed Muka et al. (37) steps 19 and 23. A random-effects meta-analysis was performed when data were sufficiently homogeneous in population, intervention, comparator, and outcome measurements. For continuous outcomes such as BMD scores, standardized or weighted mean differences (SMD/WMD) with 95% confidence intervals were calculated. For dichotomous outcomes like fracture incidence, risk ratios (RR) or odds ratios (OR) were computed as appropriate.

Two separate meta-analyses were conducted using random-effects models. The first meta-analysis included studies reporting hazard ratios (HRs) with corresponding 95% confidence intervals (35). For this analysis, log-transformation of the reported HRs was applied to stabilize variance and enable linear modeling. Standard errors (SEs) of the log(HR) values were derived from the upper and lower confidence interval limits using the following formula:


$$S\,E=\frac{l\,n\,\left(u\,p\,p\,e\,r\,b\,o\,u\,n\,d\,C\,I\right)-l\,n\,\left(l\,o\,w\,e\,r\,b\,o\,u\,n\,d\right)}{2\,x\,1.96}$$

The log(HR) values and their corresponding SEs were then pooled using an inverse-variance random-effects model, with heterogeneity estimated via restricted maximum likelihood (REML). The Hartung-Knapp method was applied to calculate more robust confidence intervals. This method is particularly suitable when a limited number of studies is included (45).

The second meta-analysis used dichotomous data on hip fracture events and total participants in exposed and unexposed groups. Individual study estimates were expressed as risk ratios (RRs), calculated using standard formulas. These RRs were then combined using an inverse-variance random-effects model. Event counts were not displayed in the forest plot, to facilitate clearer visual interpretation of effect sizes.

#### 2.1.7 Assessment of heterogeneity and sensitivity analyses

Heterogeneity was assessed using the I^2^ statistic and τ^2^ estimates (46). Given the small number of studies included in each meta-analysis and the expected methodological heterogeneity among observational designs, a random-effects model was selected *a priori*. Although statistical heterogeneity was negligible (I^2^ = 0%), the random-effects approach provides more conservative estimates, particularly under the Hartung-Knapp adjustment, which is more robust with small sample sizes (47). Fixed-effects models were not applied as the assumption of a shared true effect across diverse populations and exposure definitions was considered inappropriate.

In line with Cochrane Handbook recommendations, we conducted sensitivity analyses using three approaches: visual inspection of funnel plots, Egger’s regression test to detect small-study effects, and leave-one-out analyses to assess the robustness of the pooled estimates (35).

#### 2.1.8 Study protocol

A study protocol was written and approved by members of Working Group 3 (WG3) of the PIMENTO COST Action (CA20128–Promoting Innovation of Fermented Foods). The protocol was registered in the Open Science Framework (OSF)^1^ under the identifier doi: 10.17605/OSF.IO/Q8YZD.

### 2.2 Non-systematic components of the review

In accordance with EFSA’s scientific guidance (38, 39), we conducted non-systematic reviews on three key aspects to support the systematic review findings:

#### 2.2.1 Characterization of yogurt as the food constituent

Literature searches were conducted in PubMed and Web of Science to identify publications describing the nutritional composition, microbiological characteristics, and bioactive components of yogurt relevant to bone health. This search included terms such as “yogurt and composition,” “yogurt and fermentation,” “yogurt and bacteria,” and “yogurt and bioactive compounds.” Information was extracted on standard definitions of yogurt, bacterial strains involved in fermentation, nutritional profiles, and compounds generated during fermentation that might have an influence on bone physiology.

#### 2.2.2 Supportive evidence for biological plausibility

To establish mechanistic plausibility, we searched for *in vitro*, *ex vivo*, animal studies, and human studies investigating yogurt’s effects on bone health. Keywords included “yogurt,” combined with “bone,” “osteoblast,” “osteoclast,” “mineral absorption,” and “bone turnover.” We focused on potential mechanisms: enhanced ions bioavailability, synthesis of vitamins, gut microbiota modulation, hormone-mediated pathways, and prebiotic effects. Evidence was prioritized based on quality, relevance to humans, and consistency with clinical findings.

#### 2.2.3 Safety assessment

Adverse events reported in the included human studies were reviewed, and this was supplemented by a focused literature search on yogurt safety. Search terms included “yogurt safety,” “fermented dairy adverse effects,” and “yogurt tolerance.” Particular attention was given to potentially vulnerable populations such as those with lactose intolerance or dairy allergies. This assessment was conducted in accordance with Section 5 of the EFSA guidance on safety considerations for health claim applications (39). Finally, a wider literature search was carried out on the safety of the microorganisms used for fermentation and other possible risks of contamination during the manufacturing process.

## 3 Results and discussion

### 3.1 Identification of the pertinent human efficacy studies

**FIGURE 1:**
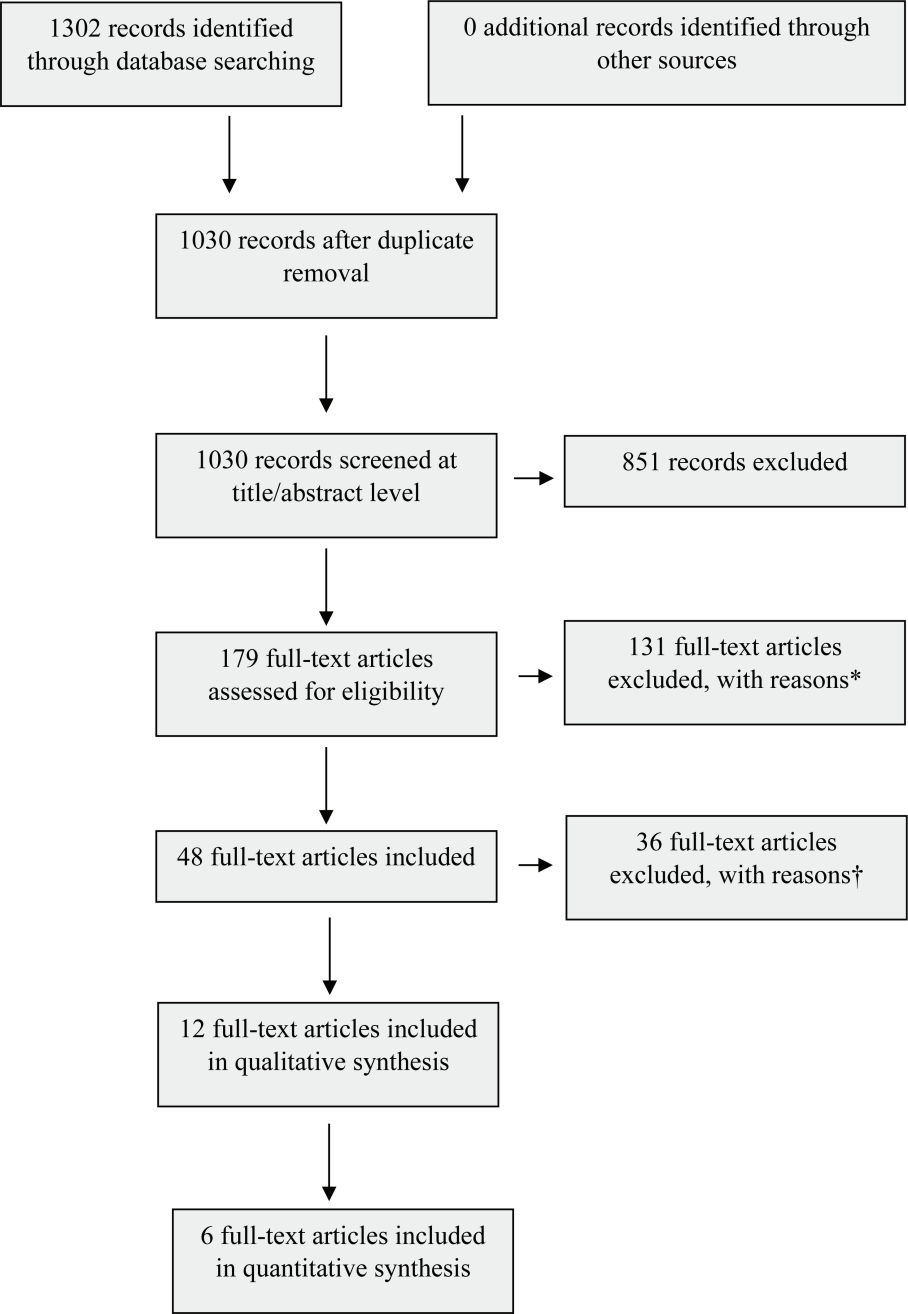
Flow diagram of database searches and results.

Observational and interventional studies, including randomized controlled trials (RCTs) and non-randomized interventions, were among our eligibility criteria and both types of studies were found in the 1302 records analyzed (Figure 1). However, after screening titles, abstracts, and full texts in CADIMA according to predefined inclusion and exclusion criteria, only 12 articles reporting observational studies, encompassing cohort, case-control and cross-sectional studies, met the established eligibility criteria and were finally selected (Figure 1). The selected studies were conducted in various adult populations including postmenopausal women (Table 2). They assessed endpoints such as BMD, bone turnover markers, and risk of fracture. We did not find any suitable randomized or non-randomized clinical trials that met the eligibility criteria.

**TABLE 2:** Characteristics of studies selected for this systematic review examining the association between yogurt consumption and bone health.

Authors (year, country)	Study design, population	Cases, *n*	Age, y	Gender F/M (%)	Diet assessment	Serving size	Consumption	Aim of study	Comparator	Bone health outcome	Duration of follow-up, years (range)
Webster et al. (26), UK	Prospective UK women’s cohort study (UKWCS), middle-aged women recruited between 1995 and 1998	26318	35–69 at baseline	F (100)	FFQ	125 g	Mean daily intake (SD) (g/day); women with hip fractures: 61.0 (67.5), Women without hip fracture: 59.6 (68.2)	Association of food, nutrients and hip fracture risk	Yogurt intake in women without hip fracture. Subgroups according to BMI < 18.5, 18.5–24.9, >25 kg/m^2^	Hip fracture	22.3
Sahni et al. (52), USA	Prospective cohort study; men and women, followed for incident of hip fracture from 1988 to 2008	764	68–96, mean 77	F, M	FFQ	1 cup	F: mean 0.57 ± 1.5 servings/wk (range 0–17.5 servings/wk) M: mean 0.20 ± 0.87 servings/wk, (range 0–7 servings/wk)	Association of dairy food intake (yogurt and cheese) with risk of hip fracture in older adults from the Framingham original cohort	No intake	Hip fracture	11.6 (0.04–21.9)
Yuan et al. (27), USA	Prospective nurses’ health study (NHS)	103003	mean age 48 y at baseline	F (100)	FFQ	1 cup (equivalent to 236.59 mL)	1–3 servings/mo, 1 serving/wk, 2–4 servings/wk, 5–6 servings/wk, 1 serving/d, 2–3 servings/d, 4–5 servings/d, or >6 servings/d	Associations between total dairy, yogurt, milk, and cheese and fragility fracture risk	<1 serving/wk as a reference	Hip fracture	No follow up
Sahni et al. (21), USA	Cross-sectional prospective Framingham offspring study	3212	26–85 mean age 55 y at baseline	F = 1681, M = 1331 (56/44)	FFQ	1 cup (8 ounces, 226.8 g)	Dairy intakes (milk, yogurt, cheese, cream) servings/wk	Association of yogurt consumption with hip fracture and BMD	Yogurt intake into three groups based on servings per week: no intake (0 servings/wk); Medium intake (≤4 servings/wk); High intake (>4 servings/wk)	Hip fracture/BMD	12–16 years follow up for hip fracture; no follow up for BMD
van Dongen et al. (51), USA	Cross-sectional prospective Framingham heart study offspring and generation	2626	32–81 mean age, 55 (F), 50 (M)	F = 1104 M = 1522 (42/58)	FFQ	1 cup	Categories: low (0–0.4 servings/wk, raw mean = 0); medium (>0.4–3.3 servings/wk, raw mean = 0.5); high intake (>3.3 servings/wk, raw mean = 3)	Association of dairy food intake with spine quantitative computed tomography bone (QCT) measures	Yogurt intake wasn’t assessed and correlated individually	BMD	No follow up
Laird et al. (19), Ireland	Cross-sectional prospective study, community dwelling older adults	4310	>60	F = 1405 M = 2905 (67/23)	FFQ	114 g	Categories: non-consumers; low consumers (2–3 times per week); high consumers (>once per day)	Associations of yogurt intakes with bone health and frailty in older adults	Consumption level: no, low, and high	BMD	No follow up
Machado-Fragua et al. (23), Spain–UK	Spain: a subset of the ENRICA study (seniors ENRICA) UK: biobank cohort study	Spain: 2981 UK: 8927	≥60	F/M	FFQ	ENRICA: serving/d (200 ml of milk, 125 g of yogurt and 40 g of cheese)	Consumption of whole milk, part-skim and skim milk, whole- and low-fat yogurt, and cheese: none, 0.5, 1, 2, 3, 4, 5, >6 servings/day	Dairy consumption and risk of falls in two European cohorts of older adults	Consumption level; lowest level as the comparator	Falls	Spain: up to 7.2 (median: 5.4) UK: up to 10.2 years (median: 3.2)
Feskanich et al. (22), USA	Nurses’ health study (NHS) and health professionals follow-up study (HPFS)	NHS: 80600 HPFS: 43306	NHS: 34–60 HPFS: 50–75	NHS: F (100) (menopause) HPFS: M (100)	FFQ	1 cup (240 mL)	Categories: never or ?1/month, 1–3/month, 1/week, 2–4/week, 5–6/week, 1/day, 2–3/day, 4–5/day, ≥6/day)	Association of milk and other dairy foods and risk of fracture in men and women in USA	Consumption level; servings per time (week/day)	Hip fracture	Mean follow-up: NHS 20.8 HPFS 17.5
Park et al. (20), Korea	Cross-sectional study; community-based cohort of the Korean genome and epidemiology study, postmenopausal women	1573	40–69	F (100)	FFQ	Yogurt intake	Categories: None; ≤2–3 times/month; >2–3 times/month; to 5–6 times/week; >5–6 times/week	Intake of dairy products and risk of osteoporosis in Korean postmenopausal women	Risk of osteoporosis between non-consumers and those with various frequencies of dairy product, milk, and yogurt intake	Osteoporosis	4
Millar et al. (50), USA	Framingham osteoporosis study	1140	64 (SD 8)	F = 652 M = 488 (57/43)	FFQ	1 cup	9 consumer categories: from <1 serving/month to >6 servings/d	Association of dairy food intake with measures (HR-pQCT tomography) of bone microarchitecture (failure load, cortical BMD, cortical thickness, trabecular BMD, and trabecular number)	Consumption in servings per week	BMD	No follow up
Kojima et al. (49), Japan	Population-based osteoporosis (JPOS) cohort study, Japanese women	1429	≥45 at baseline	F (100)	FFQ	Yogurt drinks (200 mL serving), yogurt (100 mL serving)	Categories: Seldom; 1–3 times/w; 4–6 times/w; 1 time/d; 1.5 times/d; 2 times/d; >3 times/d	Association of dairy product consumption and risk of fractures in postmenopausal women in Japan	Low or no yogurt consumption/ Moderate and high yogurt consumption	General fractures	15.1 (10.1–15.4)
Michaëlsson et al. (48), Sweden	Swedish mammography cohort study	61240 (5827 with hip fracture)	>39 (39–74 at the start)	F (100)	FFQ	200 mL	Low-fat (0.5%) and regular fat (3%) - considered together; yogurt and soured milk - reported under the same food category	Exploration of the protective effect of yogurt consumption in women	Consumption level: 0 servings per day; <1 servings per day; ≥1 to <2 servings per day; ≥2 servings per day	Hip fracture	No follow up

F, female; M, male; FFQ, food frequency questionnaire; BMD, bone mineral density.

Although 12 articles were selected, they corresponded to 14 studies, because in one article two different cohorts, one from the UK (The UK Biobank Cohort) and one from Spain (The Seniors-ENRICA Study), were reported and compared (23), and in another article two different end-points, hip fractures and BMD, were assessed independently (21). Notably, only a single study (19) specifically addressed the effect of yogurt consumption on bone health outcomes. In the remaining studies, yogurt intake was considered as part of a broader category of milk or total dairy food consumption, making it challenging to isolate yogurt’s unique impact. Five out of the 14 studies were conducted in Europe (19, 23, 26, 48), two in Asia (20, 49), and seven in the USA (21, 22, 27, 50–52). Although a majority studies were from the USA, these included populations from only two independent cohorts, The Framingham Original Cohort and its derivatives (The Framingham Offspring Study, The Framingham Heart Study, and The Framingham Osteoporosis Study) (21, 50, 52) and the Nurses’ Health Study (22, 27, 51). Of the Asian cohorts, one was from Korea (20) and the other one from Japan (49).

*Reasons:

Population-related exclusions (*n* = 28) arose from studies involving adults with pre-existing fractures at enrolment, those specifically recruiting pregnant women or infants, or animal models, which did not meet our target demographic of healthy adults or individuals with osteoporosis/osteopenia.

Intervention/exposure exclusions (*n* = 117) were primarily due to non-eligible fermented products, including alcoholic beverages exceeding 1.25% alcohol content, non-nutritional applications (e.g., topical or nasal use), or interventions containing confounders such as added prebiotics or bioactive compounds. Probiotics were excluded unless the microbial strains carried out the milk fermentation. Studies employing prebiotic-enriched foods, postbiotics, or pill-based supplements (e.g., algal extracts) were also omitted.

Outcome-related exclusions (*n* = 42) involved studies failing to report predefined bone health metrics, such as changes in BMD (e.g., osteoporosis-to-osteopenia transitions), incidence of fractures (hip, wrist, spine), serum calcium/alkaline phosphatase levels, or adverse effects per CTCAE criteria. Additionally, studies lacking valid effect measures (e.g., hazard ratios, weighted mean differences) were excluded.

Comparator-related exclusions (*n* = 32) pertained to studies without a defined control group (e.g., placebo, non-fermented food comparator).

Other exclusions (*n* = 6) included unavailable full texts (*n* = 5) and duplicate records (*n* = 1).

†Reasons:

Ineligible intervention (*n* = 25). Independent exposure to yogurt not recorded (*n* = 18): studies analyzed mixed dairy intake without disaggregating yogurt-specific effects, rendering data non-extractable for our review.

Yogurt with non-permitted additives (*n* = 7): included trials testing yogurt enriched with probiotics (*n* = 2), vitamin D (*n* = 1), or other bioactive compounds (*n* = 4), which confounded the assessment of yogurt’s intrinsic properties.

Inappropriate study design (*n* = 6): (a) non-yogurt-specific RCTs (*n* = 3): although randomized, these trials evaluated composite interventions (e.g., yogurt combined with supplements or other fermented foods) without isolated yogurt arms; (b) uncontrolled or non-comparative designs (*n* = 3): lacked placebo or fermented food comparators such as milk or milk plus vitamin D.

Irrelevant Outcomes (*n* = 5): insufficient follow-up (*n* = 4): (a) study durations < 6 months, inadequate to detect bone density or fracture risk changes; (b) on-skeletal endpoints (*n* = 1): focused on general well-being or inflammatory markers without assessing BMD, fractures, or bone-related biomarkers (e.g., serum calcium, alkaline phosphatase).

Two studies reported a statistically significant association between yogurt consumption and improvement of the endpoint under consideration, BMD and bone-associated biomarkers (19) and risk of osteoporosis (20). In the latter work, a protective effect on radius osteoporosis risk was found in high frequency yogurt consumers as compared with non-consumers [HR = 0.51, 95% CI: 0.30–0.85 for >5–6 servings/week vs. non-consumers (*P* for trend = 0.0167)] (20). Similarly, total hip and femoral neck BMD in females were 3.1%–3.9% higher among those with the highest yogurt intakes compared to the lowest (*P* < 0.05) (19). These authors further detected a positive effect in some physical function measures, such as the Timed Up and Go response (−6.7% reduction; *P* = 0.013) in high yogurt consumers. In one additional study (21), a weak association between yogurt intake and a protective effect on hip fractures was identified, although the reported Hazard Ratios was not significant [HR(95% CI): ≤4 serv/wk: 0.46 (0.21–1.03) vs. >4 serv/wk: 0.43 (0.06–3.27)]. However, a statistically significant protective effect was found for BMD at the trochanter (*P*-value for > 4 serv/wk intake vs. no intake = 0.03).

### 3.2 Meta-analysis results

#### 3.2.1 Effect of yogurt consumption on hip fracture risk

**FIGURE 2:**
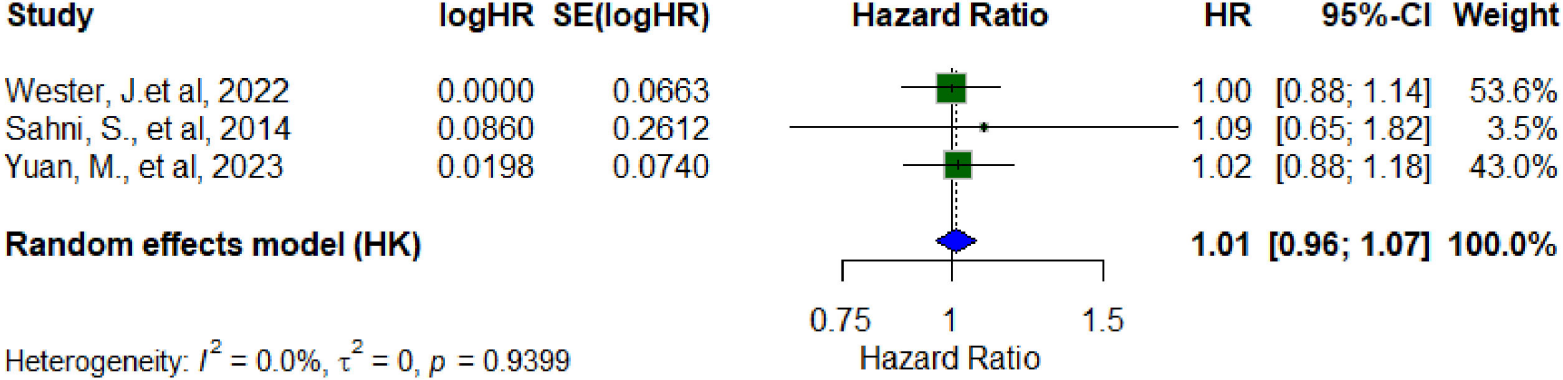
Forest plot random effect model for hazard ratio for hip fracture among cohort studies and yogurt.

The hip fracture studies comprised large population-based cohorts with extended follow-up periods: Webster et al. (26) evaluated 26,318 British women over 14 years; Sahni et al. (52) included 764 older adults from the Framingham Original Cohort with a mean follow-up of 13 years; and Yuan et al. (27) analyzed 103,003 women from the Nurses’ Health Study over a period of up to 32 years. The pooled analysis did not reveal any statistically significant association, with consistent findings reported across all studies [HR = 1.01 (95% CI: 0.96–1.07; *p* = 0.85)], indicating no statistically significant relationship between yogurt consumption and hip fracture risk at the reported intake levels (Figure 2). No heterogeneity was observed among the studies (I^2^ = 0.0%, τ^2^ = 0, *p* = 0.9399). Visual inspection of the funnel plot and Egger’s test (*p* = 0.76) did not suggest evidence of publication bias (53). However, the small number of the studies (*n* = 3) limits the reliability of this assessment.

#### 3.2.2 Effect of yogurt consumption on BMD

**FIGURE 3:**
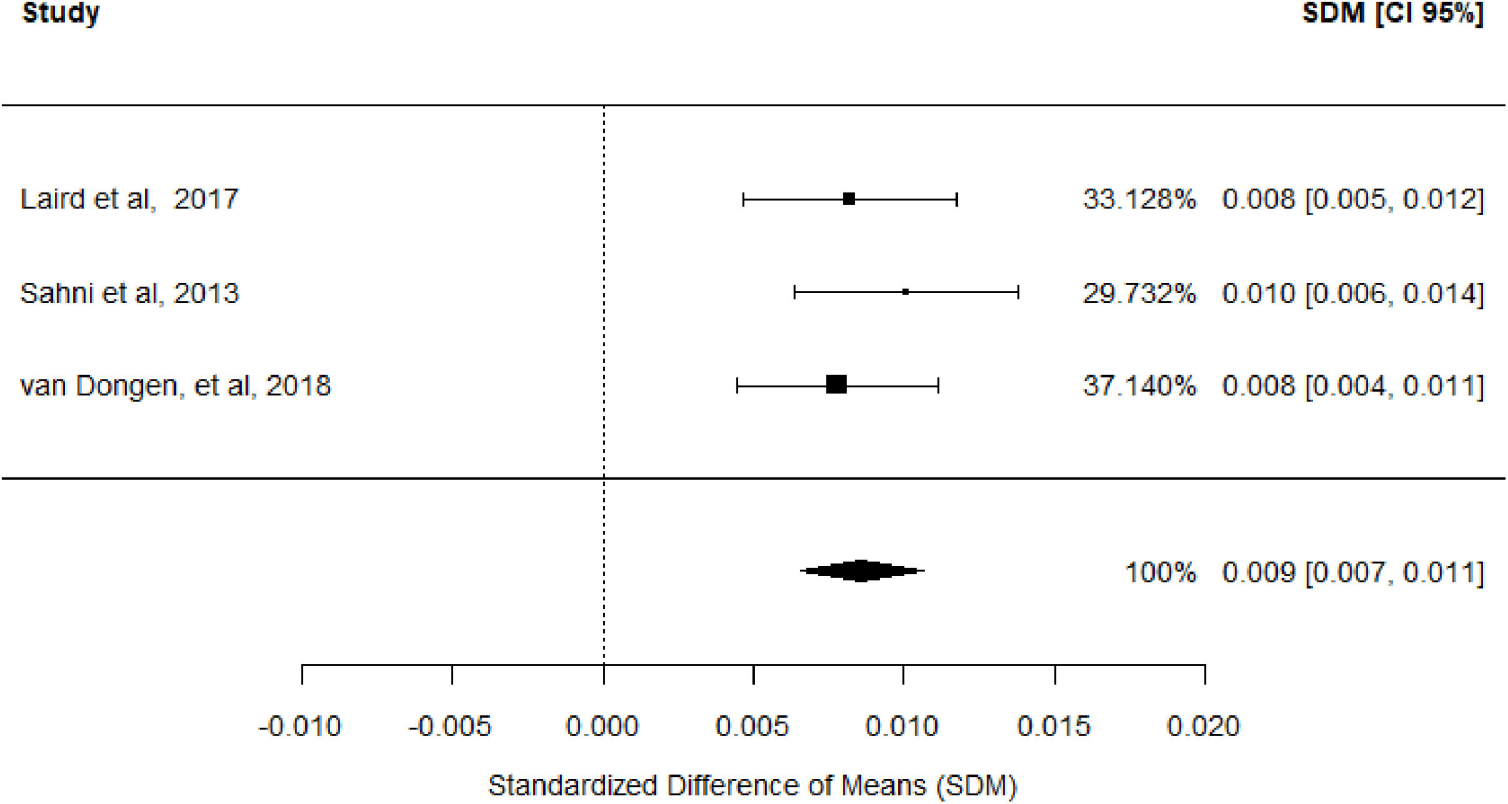
Forest plot of the association of yogurt consumption and femoral BMD.

Three studies were included to assess the effects of yogurt consumption on femoral BMD, as measured by dual-energy X-ray absorptiometry. The random-effects model demonstrated a statistically significant positive effect (standardized mean difference [SMD] = 0.009; 95% CI: 0.007–0.011), with complete homogeneity across studies (I^2^ = 0%) (Figure 3). However, the magnitude of the effect was clinically negligible (SMD < 0.2), suggesting a minimal practical impact on skeletal health. The funnel plot displays SMD for the three included studies. In the absence of publication bias, studies should be distributed symmetrically about the pooled effect estimate. Egger’s test [Intercept: 0.008 (95% CI: −0.015 to 0.0310; *p*-value: 0.423] indicates no statistically significant evidence of small-study effects.

### 3.3 Quality and bias of human studies

The studies assessing hip fracture demonstrated high methodological quality in the Newcastle Ottawa Scale (NOS), supported by well-defined cohorts, extended follow-up, and robust outcome validation (Tables 3, 4). Overall, the methodological quality of the included evidence was deemed satisfactory to support the conclusions of the meta-analysis. Studies differ widely in number of individuals followed (from 764 up to 103,003), male/female sex ratio (with five studies considering only women), mean or range age, endpoints (general fractures, hip fracture, falls, osteoporosis, BMD, and bone-associated biomarkers), duration of the follow up period (from one-point analysis up to 20.08 years), and yogurt consumption (both in serving size and servings per time unit). These large differences make difficult a comparison of the results from the evaluated studies.

**TABLE 3:** Characteristics of the selected studies.

No. of individuals	Sex	Age (mean/range)	Endpoint	Mean follow-up duration	Fractures-cases	Yogurt consumption	Final output	References
	**F/M (%)**							
26,318	100/0	35–69 years	Hip fracture	Median 22.3 years	822	59.6 ± 68.2 g/day	Neutral^a^	Webster et al. (26)
2,981	55/45	>60 years	Falls	5.4 years	801	73.5 ± 82.5 g/day	Neutral	Machado-Fragua et al. (23)
8,927	50/50	>60 years	Falls	3.2 years	201	51.25 ± 56.25 g/day	Neutral	Machado-Fragua et al. (23)
123,906	65/45	>50 years	Hip fracture	20.08 years	2,832	1% category 240 mL/d	Neutral	Feskanich et al. (22)
4,31	67/23	73.1 years	BMD^b^/biomarkers	–	–	50.16 (0–120) g/day	Positive	Laird et al. (19)
1,573	100/0	58 (40–69) years	Osteoporosis	3.4 years	273	27.9 g/day	Positive	Park et al. (20)
1,226	57/43	64 years	BMD	–	–	340/180 g/week	Neutral	Millar et al. (50)
1,429	100/0	>40 (63.3) years	General fractures	15.1 years	172	Servings/week^c^	Neutral	Kojima et al. (49)
38,071	100/0	54 years	Hip fracture	22 years	5,827	100 g/week	Neutral	Michaëlsson et al. (48)
764	w/m	77 (68–96) years	Hip fracture	11.6 years	97	80 ± 140 g/week	Neutral	Sahni et al. (52)
3,212	56/44	55 (26–85) years	Hip fractures	12 years	43	172 ± 392 g/week	Weak	Sahni et al. (21)
2,506	56/44	55 (26–85) years	BMD	–	–	172 ± 392 g/week	Weak	Sahni et al. (21)
2,626	42/58	50 (32–81) years	BMD	–	–	280/160 g/week	Neutral	van Dongen et al. (51)
103,003	100/0	48 years	General factures	24 years	5,495	? Servings/week	Neutral	Yuan et al. (27)

*^a^*Neutral means that not statistically significant association between yogurt consumption and a reduction or increasing of the considered endpoint was observed; positive means statistically significant association between yogurt consumption and reduced/enhanced endpoint; weak means that there was a trend between yogurt consumption and a reduction or increasing the endpoint but without statistical significance.

*^b^*BMD, bone mineral density.

*^c^*Servings range from 200 to 236 mL.

**TABLE 4:** Application of the Newcastle-Ottawa quality assessment scale for cross-sectional to the selected cohort studies.

Selection (maximum 5 stars)				Comparability (Maximum 2 stars)	Outcome (maximum 3 stars)			References
**Representative population**	**Non-exposed cohort**	**Ascertainment of exposure**	**Outcome at the start**	**Design and analysis**	**Assessment of outcome-statistical test**	**Follow up long enough for outcome to occur**	**Adequacy of follow up of cohorts**	
Yes (**)	Same community (*)	Validated FFQ (**)	No	Good (**)	Self-report^a^ (**)	Yes (22.3 years)	Yes	Webster et al. (26)
Yes (**)	Same community (*)	Validated FFQ (**)	No	Good (**)	Self-report (**)	Yes (7.2 years)	Yes	Machado-Fragua et al. (23)
Yes (**)	Same community (*)	Validated FFQ (**)	No	Good (**)	Self-report (**)	Yes (3.2 years)	Yes	Machado-Fragua et al. (23)
Yes (**)	Same community (*)	Validated FFQ (**)	No	Good (**)	Self-report^b^ (***)	Yes (3.2 years)	Yes	Feskanich et al. (22)
Yes (**)	Same community (*)	Validated FFQ (**)	No	Good (**)	Validated self-report^c^ (**)	Yes (3.2 years)	Yes	Laird et al. (19)
Somewhat (*)	Same community (*)	Validated FFQ (**)	Yes	Good (**)	Independent (***)	No follow up	–	Park et al. (20)
Somewhat (*)	Same community (*)	Validated FFQ (**)	Yes	Good (**)	Independent (***)	Yes (4.2 years)	Yes	Millar et al. (50)
Somewhat (*)	Same community (*)	Validated FFQ (**)	Yes	Good (**)	Independent (***)	No follow up	–	Kojima et al. (49)
Yes (**)	Same community (*)	Validated FFQ (**)	Yes	Good (**)	Self-report	Yes (15.1 years)	Yes	Michaëlsson et al. (48)
Somewhat (*)	Same community (*)	Validated FFQ (**)	Yes	Good (**)	Independent (***)	No follow up	–	Sahni et al. (52)
Somewhat (*)	Same community (*)	Validated FFQ (**)	No	Good (**)	Independent (***)	Yes (12 years)	Yes	Sahni et al. (21)
Somewhat (*)	Same community (*)	Validated FFQ (**)	No	Good (**)	Self-report (**)	Yes (12 years)	Yes	Sahni et al. (21)
Somewhat (*)	Same community (*)	Validated FFQ (**)	No	Good (**)	Independent (***)	No follow up	–	van Dongen et al. (51)
Somewhat (*)	Same community (*)	Validated FFQ (**)	Yes	Good (**)	Independent (***)	No follow up	–	Yuan et al. (27)

*^a^*The study did not differentiate between fragility and traumatic fractures.

*^b^*Validated through medical records in a small set.

*^c^*Higher yogurt consumers were also taking vitamin D.

### 3.4 Certainty of evidence assessment (GRADE)

The certainty of the evidence was assessed using the GRADE approach, following Cochrane guidance (35, 44). The two outcomes under evaluation, hip fracture and BMD, were graded separately, and the results summarized in Table 5. As all studies were observational in design, the initial certainty rating for each outcome began at “low.” We evaluated upgrading criteria, including large magnitude of effect, presence of a dose-response gradient, and potential residual confounding. None of these criteria were met: the size effect was small (e.g., SMD = 0.009 for BMD; HRs close to 1.0 for hip fracture), no dose-response relationships were observed, and confounding factors were well controlled in most studies. Therefore, no upgrading was applied. We also assessed the five downgrading domains: risk of bias: overall low, with average NOS scores between 7.0 and 8.3; inconsistency: none; results were consistent across studies (e.g., I^2^ = 0%); indirectness: no serious concerns; populations, exposures, and outcomes were relevant; imprecision: confidence intervals were relatively narrow; publication bias: not suspected.

**TABLE 5:** GRADE assessment of studies included in quantitative analysis.

Fermented food	Mortality outcome	Direction of effect	Strength of evidence	Avg. NOS score	No. of studies	Total sample size	HR/SMD range	Avg. CI width	Follow-up (years)	Geographic region	Key notes
Yogurt	Hip fracture	No effect	Low	8.3	3	∼130,000+	HR: 1.00 (0.88–1.14) to 1.09 (0.65–1.82)	∼0.48	13–32	USA, UK	Consistent and null results; no heterogeneity (I^2^ = 0%); large cohort with good multivariate fit
Yogurt	Femoral BMD	Slight increase	Low	7.0	3	∼7,900+	SMD: 0.0086 (0.0056–0.0116)	∼0.006	N/A (cross-sectional)	USA, Ireland, Netherlands	Small but significant effect; DXA/QCT measurements; cross-sectional analyses with good control for confounding

Our systematic review and meta-analysis found no significant association between yogurt consumption and hip fracture risk, based on data from large, long-term cohort studies. The pooled estimates were consistent across populations and showed hazard ratios close to unity. For BMD, the evidence indicated a small but statistically significant positive effect. However, the magnitude of this effect was clinically negligible. Overall, the aggregated findings suggest that yogurt consumption is not convincingly linked to fracture prevention and, at best, has a marginal impact on BMD. Therefore, based on these assessments, the certainty of the evidence for the two outcomes, hip fracture and BMD, remained low.

### 3.5 Mechanism of action

According to international standards, to ensure the functional properties of yogurt, viable counts of the two lactic acid bacteria (LAB) species in yogurt, *S. thermophilus* and *L. bulgaricus* must reach at least 10^7^ colony-forming units (cfu) per gram at the end of shelf life (≈28 days). However, none of the studies included in this review reported bacterial counts or verified the microbial viability in the yogurts consumed, limiting the ability to assess the potential contribution of the live cultures to the observed outcomes.

The mechanisms through which yogurt could support bone health may involve both nutritional, biochemical, and microbiological pathways. During fermentation, *S. thermophilus* and *L. bulgaricus* metabolize lactose into lactic acid, decreasing pH and enhancing calcium solubility, which improves intestinal absorption of this mineral (54). Acidification not only facilitates passive diffusion but may also stimulate active transport through the upregulation of expression of calcium-binding proteins.

Yogurt is a natural source of essential micronutrients, including vitamins B_2_, B_6_, B_12_, and K_2_, which serve as cofactors in osteoblast differentiation, collagen synthesis, and the γ-carboxylation of osteocalcin –crucial for bone matrix mineralization (55). Of particular interest is vitamin K_2_ (menaquinone), which plays a key role in calcium metabolism and bone strength (17). The specific isoform of vitamin K_2_ (e.g., MK-4 to MK-13) present in yogurt varies depending on the starter cultures and fat content of the milk used for production (56), which may partially explain the heterogeneity in outcomes across studies. Further, recent findings suggest a synergistic effect between vitamins K_2_ and D_3_ in the regulation of calcium homeostasis and bone formation (57).

*S. thermophilus* and *L. bulgaricus* possess proteolytic systems capable of breaking down milk proteins into peptides and amino acids with potential biological activity, including anti-inflammatory and antioxidant effects (58, 59). Additionally, these bacteria can synthesize extracellular polysaccharides (EPS) and other fermentation-derived compounds that have been linked to improved gut barrier function and systemic immunomodulation (3, 4).

Finally, strains of LAB species, including those of *S. thermophilus* and *L. delbrueckii*, can modulate the gut microbiota, promoting the production of short-chain fatty acids (SCFAs), such as acetate, butyrate, and propionate. SCFAs have demonstrated anti-inflammatory effects and the capacity to inhibit osteoclast differentiation, thereby reducing bone resorption (60). The gut–bone axis, mediated by immune signaling and cytokine modulation, further supports the potential of yogurt-derived microbiota interactions to influence systemic bone metabolism (61).

### 3.6 Bioavailability of bioactive compounds

Fermented dairy products such as yogurt may enhance the bioavailability of specific nutrients and bioactive compounds through modifications induced by fermentation (62). Compared to non-fermented milk, yogurt exhibits improved nutrient solubility, enzymatic liberation of active compounds, and interaction with gut microbiota–all of which may facilitate systemic absorption and delivery to bone tissue.

1. Calcium

Yogurt’s acidic pH, resulting from lactic acid production during fermentation, increases calcium ionization and its passive diffusion across intestinal membranes (21, 63). Some studies indicate that yogurt consumption leads to higher calcium uptake when compared to milk, suggesting that fermentation enhances mineral bioavailability (64). Increased calcium uptake from fermented milk is further supported by *in vitro* assays using Caco-2 cells (65). In addition, the favorable calcium-to-sodium ratio in yogurt may further support its efficient utilization, minimizing the risk of phosphate imbalance often associated with high-sodium dairy products.

2. Protein and amino acids

Proteins and peptides in yogurt are partially hydrolyzed during fermentation by the proteolytic systems of *S. thermophilus* and *L. bulgaricus*, resulting in the release of amino acids and short peptides (2, 58, 66). This hydrolysis may improve intestinal absorption, thereby facilitating the availability of essential amino acids to peripheral tissues such as bone. In particular, branched-chain amino acids (BCAAs) may contribute to musculoskeletal integrity. However, direct evidence on the transport and utilization of these peptides by bone cells remain limited in the reviewed studies.

3. Vitamins (B_2_, B_12_, K_2_, and D)

Yogurt can serve as a dietary source of vitamin B_2_ (riboflavin) and B_12_ (cobalamin), both of which may be synthesized or preserved during fermentation depending on the starter strains (67, 68). Although the specific mechanisms by which these vitamins contribute to bone metabolism are not fully elucidated, their increased content and stability in yogurt matrices may enhance systemic availability, particularly in populations at risk of deficiency (69).

Vitamin K_2_ (menaquinone) bioavailability also appears to benefit from the dairy matrix. Although variability in content across different yogurt types due to differences in starter cultures and fat content, some forms, such as MK-7, demonstrate more efficient intestinal absorption when delivered via yogurt (56). The interplay between vitamin D and K_2_, noted in the mechanistic studies, may be partly mediated by their co-delivery in fermented dairy products, supporting their concurrent availability for the regulation of calcium uptake (57).

4. Fermentation and gut interactions

Beyond the liberation of nutrients, the yogurt fermentation process itself may influence bioavailability via gut-level interactions. Some studies report that yogurt consumption modulates gut microbiota composition, promoting the production of short-chain fatty acids (SCFAs) (70), which may enhance nutrient absorption and barrier function. Yogurt intake has also been associated with lower serum levels of tartrate-resistant acid phosphatase 5b (Trap 5b), a marker of bone resorption (19). While anti-inflammatory effects are often discussed, their role in facilitating systemic transport of bone-relevant compounds remains an emerging area of research and was not fully explored in the reviewed articles.

### 3.7 Characterization of yogurt in the studies and its bioactive compounds

The characterization of the yogurts in the clinical and observational studies included in this review was generally very limited. In most cases, yogurt consumption was assessed through self-reported FFQs or dietary recalls, with no detailed specification regarding the physicochemical or microbiological properties of the products consumed. Only a few studies explicitly distinguished between yogurt types, such as plain versus flavored (22, 23, 48). However, even in these cases, further compositional details –such as fat content (whole, semi-skimmed, or skimmed), added sugars, or presence of fortifying agents (e.g., calcium or vitamin D)– were rarely reported (Supplementary Table 2). Similarly, none of the studies provided information on whether the yogurt was pasteurized after fermentation (a process allowed in certain countries), whether it contained probiotic strains of *Bifidobacterium* or other LAB species (also allowed in certain regions), or on the viability and concentration of the manufacturing cultures (Supplementary Table 2).

This insufficient characterization constrains the interpretability of the findings and the reproducibility of the observed associations. Given that the bioavailability and functionality of yogurt-derived nutrients and bioactive compounds –such as vitamins K_2_, B_12_, peptides, or short-chain fatty acids– are strongly influenced by fermentation processes, strain selection, and matrix composition, the lack of details limits the ability to attribute specific effects to yogurt consumption *per se*. Moreover, a limited product definition introduces exposure heterogeneity, which may partially account for the variability on the observed outcomes across cohorts.

### 3.8 Safety

Although yogurt is generally regarded as safe, the evidence base remains limited for specific populations (71). Most studies did not stratify results by lactose intolerance status, and no study systematically evaluated safety outcomes in individuals with dairy protein allergies or gastrointestinal disorders such as irritable bowel syndrome (IBS). Among the reviewed studies, only two reported to have taken into consideration possible adverse effects associated with yogurt consumption (48, 49). These effects were minor –mainly abdominal bloating and self-reported lactose intolerance– and no study attributed adverse events to the milk fermentation processes, the bacterial strains used as starters, or to the pH changes occurring during yogurt manufacture. In addition, yogurt is generally considered microbiologically stable due to its production from heat-treated milk, refrigerated storage, and because it contains a competitive microbiota that acidify the product through the synthesis of organic acids (71, 72).

### 3.9 Comparison with existing evidence from systematic reviews

The results of this systematic review partially agree with findings of previous reviews but also offer important nuances that enrich the current understanding of the role of yogurt in bone health. Similar to what Ong et al. (32) reported in their systematic review and meta-analysis, positive associations were identified in this work between frequent yogurt consumption and higher BMD or a lower incidence of hip fracture in postmenopausal women. However, while Ong and co-workers reported a significant 24% reduction in the risk of hip fracture (RR = 0.76; 95% CI: 0.63–0.92), the meta-analysis performed in this review, based on three large cohorts with long follow-ups and no heterogeneity between studies, did not find a statistically significant association (HR = 1.01; 95% CI: 0.96–1.07; *p* = 0.85). This discrepancy may be explained, at least in part, by differences in the definition of exposure (e.g., >0 vs. ≥5 times/week vs. ≥2 times/day), cohort inclusion criteria, and adjustment for relevant covariates such as physical activity, nutritional status, or intake of supplements, as well as by possible differences in the compositional characteristics of the yogurts included in the studies.

A key methodological difference between the two reviews lies in their focus on dietary exposure. While Ong et al. (32) exclusively included studies that distinguished yogurt and cheese as independent exposures, in this review only one study assessed specifically the effect of yogurt alone (19). In all others, yogurt was considered within the broad category of “dairy.” This lack of exposure specificity likely contributes to the attenuation of the effect observed in the meta-analysis. Furthermore, the review presented here focused exclusively on hip fracture as a clinical outcome, while Ong et al. (32) also considered BMD, T-score, and bone turnover markers, which might have allowed them to capture potential effects at earlier stages of bone deterioration.

Both this review and previous ones agree in pointing out the critical lack of high-quality randomized controlled trials (RCTs) evaluating the effect of a nutritionally and microbiologically well-characterized yogurt as specific dietary interventions on fracture outcomes. The few available RCTs, such as those reviewed by Ong et al. (32) are of short duration (<2 months), with intermediate outcomes (biochemical markers), and lacking statistical power to assess fractures. This methodological limitation prevents establishing robust causal relationships and underscores the urgent need for well-designed intervention studies with clear definitions of exposure and follow-up of relevance to clinical events. Studies with a duration shorter than 1 year are not considered reliable (38, 39) and were not included in our systematic review.

The systematic review and meta-analysis by Malmir et al. (73) included 34 studies (15 on osteoporosis and 21 on hip fracture) and performed separate analyses by study design. In cohort studies, no significant association was found between dairy product consumption and the risk of osteoporosis (RR = 0.82; 95% CI: 0.56–1.18) or hip fracture (RR = 0.90; 95% CI: 0.73–1.11), which is consistent with the results of our review. However, in case-control studies, yogurt consumption was associated with a 25% reduction in the risk of hip fracture (RR = 0.75; 95% CI: 0.57–0.99), suggesting that the observed effects could be influenced by selection or recall biases inherent to the study design. These authors also highlighted the heterogeneity in the definition of exposure and the lack of standardization in the portions and types of dairy products evaluated, a limitation that also affects the present review. However, the current systematic review incorporates dedicated sections aligned with EFSA guidance –such as characterization of the yogurt products, discussion of bioavailability of bioactive compounds, and mechanistic pathways– which may help to contextualize the observed associations and facilitate future standardization efforts in this research field.

Another aspect shared by all reviews is the lack of systematic assessment of adverse effects associated with yogurt consumption. This omission is particularly relevant considering the variability in the content of added sugars, saturated fats, and additives in commercial products, as well as potential intolerance to lactose or specific proteins such as A1 β-casein (55). The digestion of this casein isoform can generate bioactive peptides causing gastrointestinal pain or discomfort.

Finally, all reviews agree in pointing out the ambiguity in the definition of “yogurt” as a cross-sectional limitation. There is no systematic distinction between plain, Greek, probiotic-containing, sweetened, or fortified yogurt, which makes comparison of the results across studies and interpretation difficult. This lack of standardization, coupled with the conceptual breadth of the “dairy” category, dilutes the specific effect of yogurt and limits the applicability of the findings to produce dietary recommendations. In this sense, this review provides a more conservative and specific analysis of yogurt as a dairy product and highlights the need to move toward a precise characterization of the foods evaluated in nutritional studies.

## 4 Conclusion

The evidence from both systematic and non-systematic parts of this review was evaluated according to Section 5 of the EFSA guidance (Overall summary of pertinent scientific data), particularly according to Sections 5.2.1 (Substantiation of a causal relationship between the consumption of the food/constituent and the claimed effect) and 5.2.2 (Characterization of the relationship between the consumption of the food/constituent and the claimed effect) (39). Overall, most studies conclude on a positive trend of yogurt consumption in bone health, including the risk of hip, osteoporosis and increase of bone-associated biomarkers. However, the evidence is insufficient to establish a cause-and-effect relationship. Yogurt does not provide convincing benefits in fracture prevention, and its impact on BMD is, at best, marginal. High-quality RCTs are needed to confirm whether specific subgroups –such as individuals with low calcium intake or poor baseline bone health– might derive clinically meaningful benefits from regular yogurt consumption.

To advance in the field, future trials should employ rigorous randomized controlled designs targeting clinically vulnerable populations. For example, a double-blind RCT comparing daily yogurt intake to placebo or non-fermented dairy over at least 12 months –the smallest intervention time considered by EFSA guidelines (38) – in postmenopausal women with osteopenia could assess changes in BMD (via DXA) and fracture incidence as primary outcomes. Nutritional characterization of yogurt, microbiota profiling and dietary markers should be integrated to explore the mechanistic mode of action and the individual variability in the response.

Yogurt has demonstrated a favorable safety profile, with rare and mild adverse effects. No population with absolute contraindications was identified, although the evidence remains insufficient to evaluate risks from extreme intake or atypical formulations. From a public health perspective, these findings do not currently support the promotion of yogurt as a stand-alone intervention for fracture prevention or osteoporosis management. However, its favorable safety profile, widespread acceptability, and potential synergistic effects with other dietary components justify its further exploration in clinical evaluations; particularly, in the context of personalized nutrition and cost-effective dietary strategies.

According to the EFSA evidence grading, the overall strength of the evidence was rated as “neither convincing nor sufficient” (39), reflecting the combination of statistical consistency of observational findings only, the minimal effect size observed for BMD, the absence of significant fracture risk reduction, and the lack of RCT studies isolating yogurt as the active dietary component.

### 4.1 Limitations

This review has several limitations, primarily due to the observational nature of the included studies, which inherently carry risks of residual confounding and selection bias. The hip fracture meta-analysis was limited to only three studies, reducing statistical power and precluding meaningful subgroup analysis.

The variability in yogurt characterization across studies (e.g., dry matter, fat content, etc.) limits the capability to perform subgroup or dose-response analyses, which may be clinically relevant to identify more effective yogurt types, probiotic formulations, or consumption patterns for bone health outcomes. Addressing these gaps in future studies would enhance both the clinical interpretability and mechanistic understanding of the observed associations. These limitations further impede the mechanistic interpretation of the findings and the ability to identify potentially more effective yogurt formulations for bone health. While a statistically significant association was observed for BMD, the magnitude of the effect (SMD = 0.009) falls below clinical thresholds relevant for osteoporosis management or fracture prevention.

### 4.2 Data gaps and outlook

A critical gap identified in this review is the complete absence of RCT studies meeting the eligibility criteria. While the observational studies included provide suggestive evidence of a neutral to marginally positive association between yogurt consumption and bone health, they are insufficient to establish causality, which is pivotal to inform clinical or policy guidelines (74). This lack of trial-based evidence is especially relevant in the context of emerging fields such as nutrieconomics and nutrigenomics. Nutritional interventions –such as consumption of fermented dairy products– are increasingly being evaluated not only for their biological efficacy but also for their cost-effectiveness and potential for personalized implementation. As highlighted by Vélez-Cuellar et al. (75), nutrieconomic approaches are essential for informing public health strategies in resource-limited settings, especially regarding the prevention of chronic disease in older populations. Similarly, the work of Kassem et al. (76) underscores the potential of nutrigenomics and microbiome modulation in shaping personalized nutrition strategies. Fermented foods like yogurt can play a key role in this interface by influencing gut microbiota and interacting with host genetic pathways involved in bone and metabolic health. However, these mechanistic insights remain largely unexplored in clinical trials focusing on skeletal outcomes.

To advance the field, future research should prioritize the design and conduct of high-quality RCTs with the following features:

- Adequate duration and power: Trials should last long enough to influence clinically relevant outcomes such as BMD or fracture incidence, not only short-term biochemical markers.
- Standardized reporting: Protocols should be pre-registered and made publicly available in platforms such as ClinicalTrials.gov, OSF, or other open science repositories, with subsequent adherence to CONSORT reporting guidelines.
- Population diversity: Trials should recruit participants from different regions and ethnic backgrounds to ensure external validity, given geographic variability in diet, microbiota, and baseline risk of osteoporosis and fractures.
- Detailed characterization of yogurt products: Even if international homogenization is challenging, yogurts can and should be characterized microbiologically (e.g., strain sequencing, microbial load) and nutritionally (e.g., fat content, protein-to-calcium ratio, vitamin D fortification). This would allow meaningful comparisons across countries (e.g., Colombia vs. China) and across product types.
- Differentiation of yogurt categories: Trials should clearly distinguish between plain, Greek, Turkish, drinkable, fortified, probiotic-enriched, and “spoonable” yogurts, since their composition and microbial profiles vary considerably and may have differential effects on bone health.
- Integration of broader frameworks: Inclusion of nutrieconomic evaluations (cost, accessibility, and measurable outcomes such as fracture reduction or BMD gain) and nutrigenomic approaches (variability in response based on genetic and microbiome profiles).

Addressing these gaps will provide more robust causal evidence and help move from observational associations toward evidence-based dietary recommendations that can inform both clinical practice and public health strategies.

## Data Availability

The original contributions presented in this study are included in the article/Supplementary material, further inquiries can be directed to the corresponding authors.

## Footnotes

1
https://osf.io/

2
https://osf.io/zu4wb/
