# Supplementary material for: Impact of yogurt consumption on bone health markers in adults with or without osteoporosis: a systematic review and meta-analysis
Source: Front Nutr. 2025 Sep 30;12:1660505. doi: 10.3389/fnut.2025.1660505 (PMC12518318; doi:10.3389/fnut.2025.1660505)
Supplement: Supplementary file 2 [file Table_2.docx]

**Table S2.** Fermentation-related characteristics of yoghurt in included studies.

| **Study (Author, Year)** | **Country** | **Yoghurt type** | **Bacterial strains** | **Fermentation process (time/temp)** | **Final pH** | **Pasteurised after fermentation** | **Other notes** |
| --- | --- | --- | --- | --- | --- | --- | --- |
| Webster et al., 2022 | UK | Commercial plain yogurt | Not reported | Not reported | Not reported | Not reported | FFQ assessed frequency; no brand or specs |
| Sahni et al., 2013 & 2014 | USA | Yogurt (unspecified type) | Not reported | Not reported | Not reported | Not reported | FFQ-based intake; commercial yogurt assumed |
| Yuan et al., 2023 | USA | Low-fat (%?) yogurt | Not reported | Not reported | Not reported | Not reported | Included in multi-item FFQ |
| Van Dongen et al., 2018 | USA | Fermented dairy (yogurt included) | Not reported | Not reported | Not reported | Not reported | Category includes yogurt and other products |
| Laird et al., 2019 | Ireland | Commercial yogurt | Not reported | Not reported | Not reported | Not reported | Association with BMD and physical function |
| Park et al., 2018 | Korea | Not specified | Not reported | Not reported | Not reported | Not reported | Dietary pattern study |
| Kojima et al., 2023 | Japan | Japanese-style yogurt | Not reported | Not reported | Not reported | Not reported | BMD via calcaneal ultrasound |
| Feskanich et al., 2018 | USA | Fermented dairy (incl. yogurt) | Not reported | Not reported | Not reported | Not reported | Focused on fermented milk & fractures |
| Machado-Fragua et al., 2020 | Spain/UK | Yogurt in dietary pattern | Not reported | Not reported | Not reported | Not reported | Outcome: frailty |
| Michaëlsson et al., 2018 | Sweden | Fermented milk (not isolated to yogurt) | Not reported | Not reported | Not reported | Not reported | No disaggregated data for yogurt |

Key of abbreviations: FFQ, Food Frequency Questionnaire; BMD, Bone Mass Density
